# Comprehensive Comparison of Three Automated 25-Hydroxyvitamin D Immunoassays with a VDSCP Traceable LC-MS/MS Method Across Clinically Relevant Decision Thresholds

**DOI:** 10.3390/nu18182986

**Published:** 2026-09-11

**Authors:** Szilvia Racz, Luca Jozsa, Amrit Pal Bhattoa-Buzas, Emese Szmik, Edit Kalina, Sandor Barath, Dora Bencze, Etienne Cavalier, Harjit Pal Bhattoa

**Affiliations:** 1 Department of Medical Imaging, Faculty of Medicine, Kalman Laki Doctoral School of the University of Debrecen, University of Debrecen, 4032 Debrecen, Hungary; lakatos.szilvia@med.unideb.hu; 2 Department of Laboratory Medicine, Faculty of Medicine, University of Debrecen, 4032 Debrecen, Hungary; jozsa.luca02@gmail.com (L.J.); bhattoa.amrit@gmail.com (A.P.B.-B.); szmik.e14@gmail.com (E.S.); ekalina@med.unideb.hu (E.K.); barath.sandor@med.unideb.hu (S.B.); bencze.dora@med.unideb.hu (D.B.); 3 Department of Clinical Chemistry, University of Liège, B-4000 Liège, Belgium; etienne.cavalier@chuliege.be

**Keywords:** comparison study, 25-hydroxyvitamin D, vitamin D metabolites, DiaSorin Liaison XL, Beckman Coulter DxI 9000, Roche Cobas e602, LC-MS/MS

## Abstract

Background/Objective: Accurate measurement of serum 25-hydroxyvitamin D (25OHD) is essential for the diagnosis and management of vitamin D deficiency. Although automated immunoassays are widely used in routine clinical practice, analytical variability between platforms may lead to inconsistent patient classification around clinically relevant decision thresholds. This study comprehensively compared three automated immunoassays (DiaSorin Liaison XL, Beckman Coulter DxI 9000, and Roche cobas e602) with a Vitamin D Standardization Certification Program (VDSCP)-traceable liquid chromatography–tandem mass spectrometry (LC-MS/MS) reference method. Methods: A total of 400 anonymized adult outpatient serum samples were prospectively collected and equally distributed across four predefined vitamin D concentration categories (<25, 25–49, 50–74, and ≥75 nmol/L). Samples were stored at −70 °C before simultaneous analysis on the three automated immunoassays and the reference LC-MS/MS platform. Method comparison included Deming regression, Bland–Altman analysis, and evaluation of diagnostic performance at clinically relevant thresholds (<25, <50, and <75 nmol/L). The effects of sample storage, vitamin D metabolites (C3-epi-25OHD_3_ and 24,25-dihydroxyvitamin D_3_), and external quality assessment (DEQAS) performance were also investigated. Results: Significant analytical differences were observed between all immunoassays and LC-MS/MS. Roche cobas e602 consistently overestimated 25OHD concentrations, resulting in reduced sensitivity but excellent specificity and positive predictive value across all clinical thresholds. Beckman Coulter demonstrated the highest sensitivity for detecting vitamin D deficiency but generated more false-positive classifications, particularly at the <75 nmol/L threshold. DiaSorin Liaison XL achieved the best overall balance between sensitivity, specificity, accuracy, and agreement with LC-MS/MS, with Cohen’s κ values ranging from 0.818 to 0.895 across decision thresholds. Higher concentrations of C3-epi-25OHD_3_ and 24,25-dihydroxyvitamin D_3_ were associated with greater positive bias in Roche measurements, while Beckman Coulter showed no significant metabolite-related interference, although the present correlation analyses could not establish independent analytical interference. DEQAS performance did not consistently reflect agreement with LC-MS/MS in patient samples. Conclusions: Despite ongoing international standardization efforts, clinically important differences remain among automated 25OHD immunoassays. Assay-specific analytical characteristics significantly influence patient classification around established decision thresholds. DiaSorin Liaison XL demonstrated the closest overall agreement with the reference LC-MS/MS method, whereas Beckman Coulter prioritized sensitivity and Roche specificity. These findings highlight the importance of method-aware interpretation of vitamin D results and support continued assay harmonization to improve comparability in routine clinical practice.

## 1. Introduction

Vitamin D is an essential regulator of calcium and phosphate homeostasis and plays a fundamental role in skeletal health. Beyond its classical endocrine actions, increasing evidence suggests that vitamin D is involved in numerous extra-skeletal physiological processes, although the clinical significance of these associations remains an area of active investigation [1,2,3,4,5,6,7,8,9,10,11,12]. In circulation, vitamin D exists predominantly as 25-hydroxyvitamin D (25OHD), which is considered the most reliable biomarker of vitamin D status because it reflects both endogenous synthesis following ultraviolet light exposure and dietary intake or supplementation [13,14,15,16].

Current international guidelines recommend measurement of serum 25OHD only in individuals at increased risk of vitamin D deficiency rather than population-wide screening [17]. Clinical decision-making relies on established concentration thresholds to identify severe deficiency, deficiency, insufficiency and sufficiency, making accurate laboratory measurement essential [18]. Small analytical differences around these clinical cut-off values may result in different patient classifications and consequently influence therapeutic decisions [19].

Several analytical methods are available for the measurement of serum 25OHD, including automated immunoassays, high-performance liquid chromatography (HPLC) and liquid chromatography–tandem mass spectrometry (LC-MS/MS) [14,20,21]. LC-MS/MS is widely regarded as the reference measurement procedure because of its superior analytical specificity and its ability to separately quantify 25OHD_2_ and 25OHD_3_ while minimizing interference from structurally related metabolites.

Despite continuous technological improvements and international standardization initiatives such as the Vitamin D Standardization Program (VDSP) and the Vitamin D External Quality Assessment Scheme (DEQAS), considerable variability still exists between commercially available immunoassays [22,23,24,25,26,27,28]. Several analytical factors contribute to this variability, including incomplete release of 25OHD from vitamin D-binding protein, differences in antibody specificity toward 25OHD_2_ and 25OHD_3_, cross-reactivity with structurally related vitamin D metabolites, and calibration differences among manufacturers [13]. These analytical discrepancies may translate into clinically meaningful differences in patient classification, particularly when measured concentrations lie close to recommended clinical decision thresholds.

Previous studies comparing automated immunoassays with LC-MS/MS have reported variable agreement between platforms [29,30,31,32,33,34,35,36,37,38,39]. However, most investigations have focused primarily on overall analytical agreement without systematically evaluating assay performance within clinically relevant vitamin D categories. Furthermore, relatively few studies have simultaneously addressed important confounding factors such as sample storage, assay performance in external quality assessment schemes, and the influence of vitamin D metabolites including C3-epi-25-hydroxyvitamin D_3_ and 24,25-dihydroxyvitamin D_3_ [28,39,40,41].

The present study was therefore designed to provide a comprehensive comparison of three widely used automated immunoassays (DiaSorin Liaison XL, Beckman Coulter DxI 9000 and Roche cobas e602) against a validated LC-MS/MS reference method. Using a balanced cohort representing clinically relevant vitamin D concentration ranges, we evaluated analytical agreement, diagnostic performance at established clinical decision thresholds, the influence of sample storage and vitamin D metabolites, and assay performance within an international external quality assessment program. Our objective was to determine how analytical differences between methods may influence the clinical classification of vitamin D status in routine laboratory practice.

## 2. Materials and Methods

### 2.1. Samples

The routine diagnostic laboratory at the Department of Laboratory Medicine, University of Debrecen, receives an average of 2000 patient samples each working day. Over the years, the number of serum 25-hydroxyvitamin D (25OHD) determinations performed at the University has increased substantially. In 2024, a total of 15,036 25OHD measurements were performed, corresponding to an average of approximately 60 tests per working day [42]. Currently, serum 25OHD concentrations are measured using the DiaSorin Liaison Total 25OHD chemiluminescent immunoassay (DiaSorin Inc., Stillwater, MN, USA) on the Liaison XL automated platform. Between 31 March and 28 August 2025, a total of 400 residual serum samples were collected based on their measured 25OHD concentrations. The samples were equally distributed into four groups (n = 100 per group): Group A, <25 nmol/L; Group B, 25–49 nmol/L; Group C, 50–74 nmol/L; and Group D, ≥75 nmol/L. The authors acknowledge that no universal consensus has yet been established regarding the thresholds defining vitamin D sufficiency. Samples from individuals younger than 18 years of age, hospitalized patients, and pregnant women were excluded. Following collection, each sample was divided into four aliquots, anonymized, and immediately stored at −70 °C until analysis in October 2025.

### 2.2. Measurement Platforms

A total of four analytical methods were used for the batch analysis of the collected samples. Serum 25OHD concentrations were measured using the Beckman Access 25(OH) Vitamin D Total assay on the DxI 9000 Access Immunoassay Analyzer (Beckman Coulter Inc., Brea, CA, USA), the Roche Elecsys Vitamin D Total III assay on the Roche cobas e602 analyzer (Roche Diagnostics GmbH, Mannheim, Germany), and the DiaSorin Liaison 25 OH Vitamin D Total assay on the Liaison XL automated platform (DiaSorin Inc., Stillwater, MN, USA), all at the Department of Laboratory Medicine, University of Debrecen, Hungary. Samples with results below the analytical measuring range, i.e., <10 (DiaSorin Liaison XL, n = 13), <11 (Beckman Coulter, n = 10) and <7.5 (Roche, n = 2) nmol/L were not included in the statistical analyses. Major features of the automated 25-hydroxyvitamin D assays included in the study are presented in Table 1.

The LC-MS/MS analysis was performed at the Department of Clinical Chemistry, University of Liège, CHU Sart-Tilman, Liège, Belgium, with a CDC Vitamin D Standardization Certification Program (VDSCP) traceable LC-MS/MS method. Serum concentrations of 25-hydroxyvitamin D_3_ (25OHD_3_), 25-hydroxyvitamin D_2_ (25OHD_2_), 3-epi-25-hydroxyvitamin D_3_ (3-epi-25OHD_3_), 3-epi-25-hydroxyvitamin D_2_ (3-epi-25OHD_2_) and 24,25-dihydroxyvitamin D_3_ (24,25OH_2_D_3_) were measured using a validated UHPLC-MS/MS method, as previously described [43]. Briefly, chromatographic separation was performed on a Nexera X2 UPLC system (Shimadzu, Kyoto, Japan) coupled to a QTRAP 6500 quadrupole-linear ion trap mass spectrometer (AB Sciex, Foster City, CA, USA), equipped with an IonDrive™ Turbo V source operating in positive atmospheric pressure chemical ionization (APCI) mode. Separation was achieved on a Kinetex pentafluorophenyl column (100 × 2.1 mm, 2.6 µm; Phenomenex, Torrance, CA, USA) using a water/methanol gradient, with both mobile phases containing 0.1% formic acid. The method allowed baseline separation of 25OHD_3_ and 25OHD_2_ from their respective epimers, as well as separation of 24,25OH_2_D_3_ from potential isobaric interferences.

Calibration was performed using an eight-point calibration curve prepared in vitamin D-free human serum, ranging from 0 to 100 µg/L for 25OHD, 0 to 50 µg/L for epi-25OHD and 0 to 20 µg/L for 24,25OH_2_D_3_. Standards were verified using NIST SRM 2972 solvent-based reference materials, and method validation was performed using NIST SRM 972a and Labquality reference materials. For sample preparation, 100 µL of serum, calibrator or quality-control material was mixed with 25 µL of 4.5% zinc sulfate solution and 100 µL of an isotopically labelled internal standard mixture in acetonitrile. After vortex mixing, samples were kept for 10 min at 5 °C to facilitate protein precipitation, centrifuged for 5 min at 13,200 rpm and 10 °C, and 150 µL of the supernatant was transferred to a 96-well plate for analysis. The injection volume was 30 µL. The method was validated against certified/reference materials and showed limits of quantification of 0.5 µg/L for 24,25OH_2_D_3_, 1.1 µg/L for 25OHD_3_ and 3-epi-25OHD_3_, and 1.7 µg/L for 25OHD_2_.

**Figure 1:**
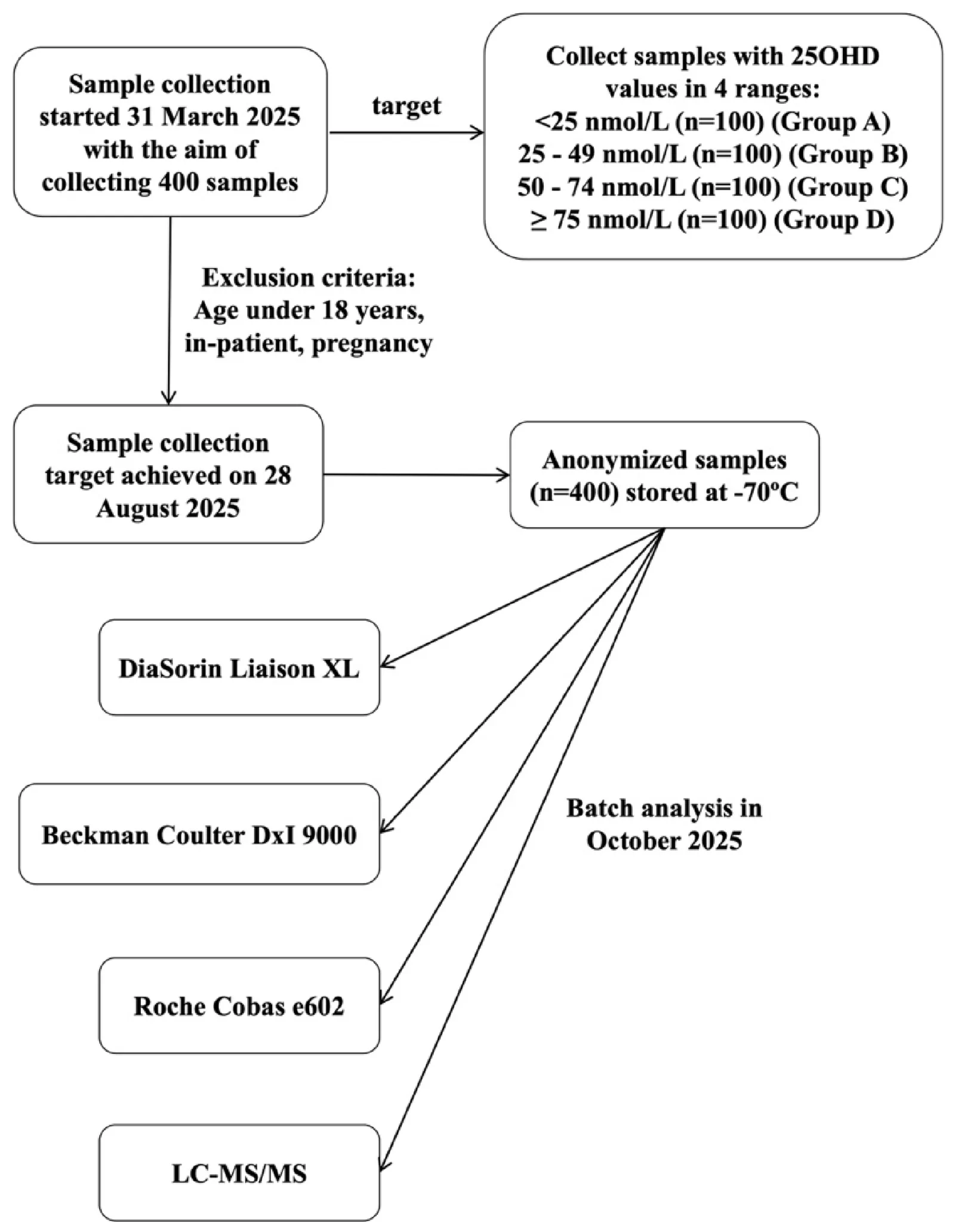
Study workflow.

Following aliquoting and storage, the frozen samples were transported from Debrecen, Hungary, to Liège, Belgium, by overnight courier on dry ice under continuous temperature monitoring to ensure sample integrity. The overall study workflow is illustrated in Figure 1.

### 2.3. External Quality Assessment (DEQAS)

To monitor analytical performance throughout the study period, a total of 15 DEQAS samples from the April, July, and October distributions were analyzed on all four analytical platforms.

### 2.4. Statistical Evaluation

Agreement between the three automated immunoassays and the reference LC-MS/MS method was evaluated using Bland–Altman analysis. Bland–Altman plots were constructed to visually assess agreement, while the mean bias and the upper and lower limits of agreement (LoA) were calculated to quantify systematic differences between methods. For all Bland–Altman analyses, the paired difference was calculated as:

Difference = Method A − Method B

For comparisons between the LC-MS/MS method and the DiaSorin Liaison XL, Beckman Coulter or Roche methods, LC-MS/MS was consistently designated as Method A. Accordingly, a positive bias indicates that the LC-MS/MS method yielded higher values than the comparator method, whereas a negative bias indicates that the LC-MS/MS method yielded lower values. For pairwise comparisons among the DiaSorin Liaison XL, Beckman Coulter and Roche methods, the method listed first in the x-axis title was consistently designated as Method A.

Deming regression analysis was performed to evaluate the relationship and determine the line of best fit between each immunoassay and the reference LC-MS/MS method. Because both platforms measured the same analyte in identical units, equal measurement-error variances were assumed, and the error-variance ratio (λ) was set to 1. Heteroscedasticity was assessed visually via scatter plots and Bland–Altman difference plots; no appreciable variation in dispersion across the measurement range was observed, justifying the use of unweighted Deming regression. Slopes and intercepts are reported alongside their two-sided 95% confidence intervals.

Diagnostic classification performance was assessed using LC-MS/MS as the reference method at the clinically relevant 25OHD thresholds of <25, <50, and <75 nmol/L. For each immunoassay, 2 × 2 contingency tables were constructed, and sensitivity, specificity, positive predictive value (PPV), negative predictive value (NPV), overall accuracy, and Cohen’s kappa coefficient were calculated. According to the conventional interpretation of Cohen’s kappa, values >0.81 indicate almost perfect agreement, while values between 0.61 and 0.80 indicate substantial agreement. Exact binomial 95% confidence intervals (CIs) were determined for sensitivity, specificity, PPV, NPV, and accuracy. Agreement between each immunoassay and LC-MS/MS was further evaluated using McNemar’s test.

All analyses were performed for the complete study cohort (n = 400) as well as for the predefined 25OHD concentration subgroups across all four analytical platforms. Normality of the paired differences was assessed using the Shapiro–Wilk test. As the data did not satisfy the assumption of normality, paired comparisons were performed using the Wilcoxon matched-pairs signed-rank test. A two-sided *p*-value < 0.05 was considered statistically significant. Statistical analyses were performed using GraphPad Prism version 9.1.2 (GraphPad Software, Boston, MA, USA).

### 2.5. Ethics Permission

The study protocol was approved by the Committee on Scientific and Research Ethics of the Hungarian Health Sciences Council (Approval No. BM/4279-3/2025).

## 3. Results

### 3.1. Effect of Storage at −70 °C

To evaluate the effect of long-term storage at −70 °C on serum 25OHD concentrations, all samples were reanalyzed on the DiaSorin Liaison XL platform. The 25OHD concentrations measured at the time of routine clinical testing (baseline) and after storage are summarized in Table 2.

As measurements on the Beckman Coulter, Roche, and LC-MS/MS platforms were performed exclusively on the stored samples, the post-storage Liaison XL results were used for all between-platform comparisons. Although storage resulted in changes in measured 25OHD concentrations for some samples, the original group allocation was retained. Consequently, samples remained assigned to their baseline categories (Group A: <25 nmol/L, Group B: 25–49 nmol/L, Group C: 50–74 nmol/L, and Group D: ≥75 nmol/L), regardless of their post-storage 25OHD concentrations.

### 3.2. 25OHD Measurement Results

**Figure 2:**
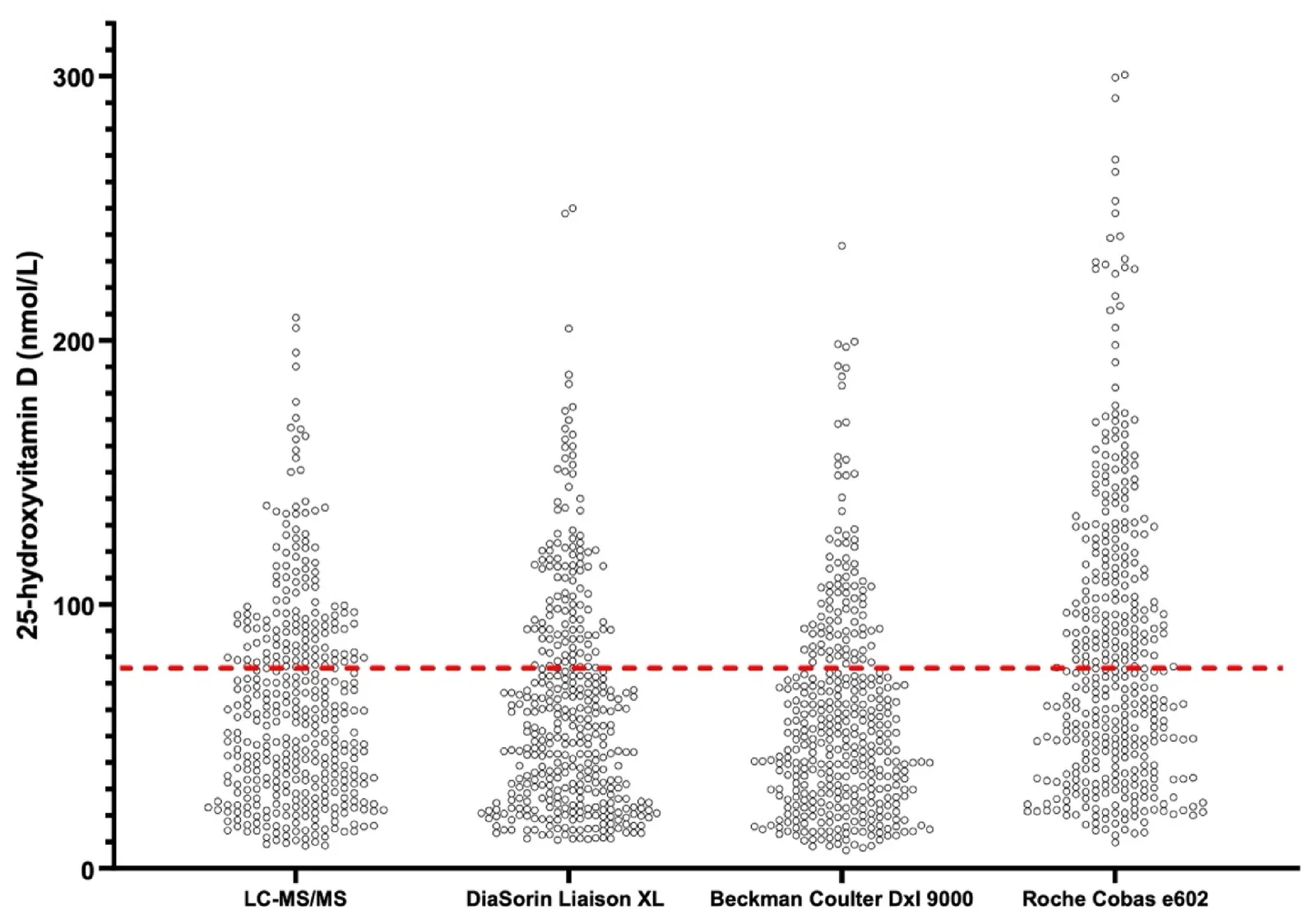
Beeswarm plots showing the distribution of serum 25-hydroxyvitamin D concentrations measured by the four analytical methods. The red dashed line represents the 75 nmol/L cut-off.

The distribution of serum 25-hydroxyvitamin D concentrations measured by the four analytical platforms is summarized in Table 3 and illustrated in Figure 2. The mean and range of the measured 25OHD concentrations for each platform are presented in Table 3, while the distribution of individual measurements is visualized using beeswarm plots in Figure 2.

### 3.3. Deming Regression and Bland–Altman Analysis

**Figure 3:**
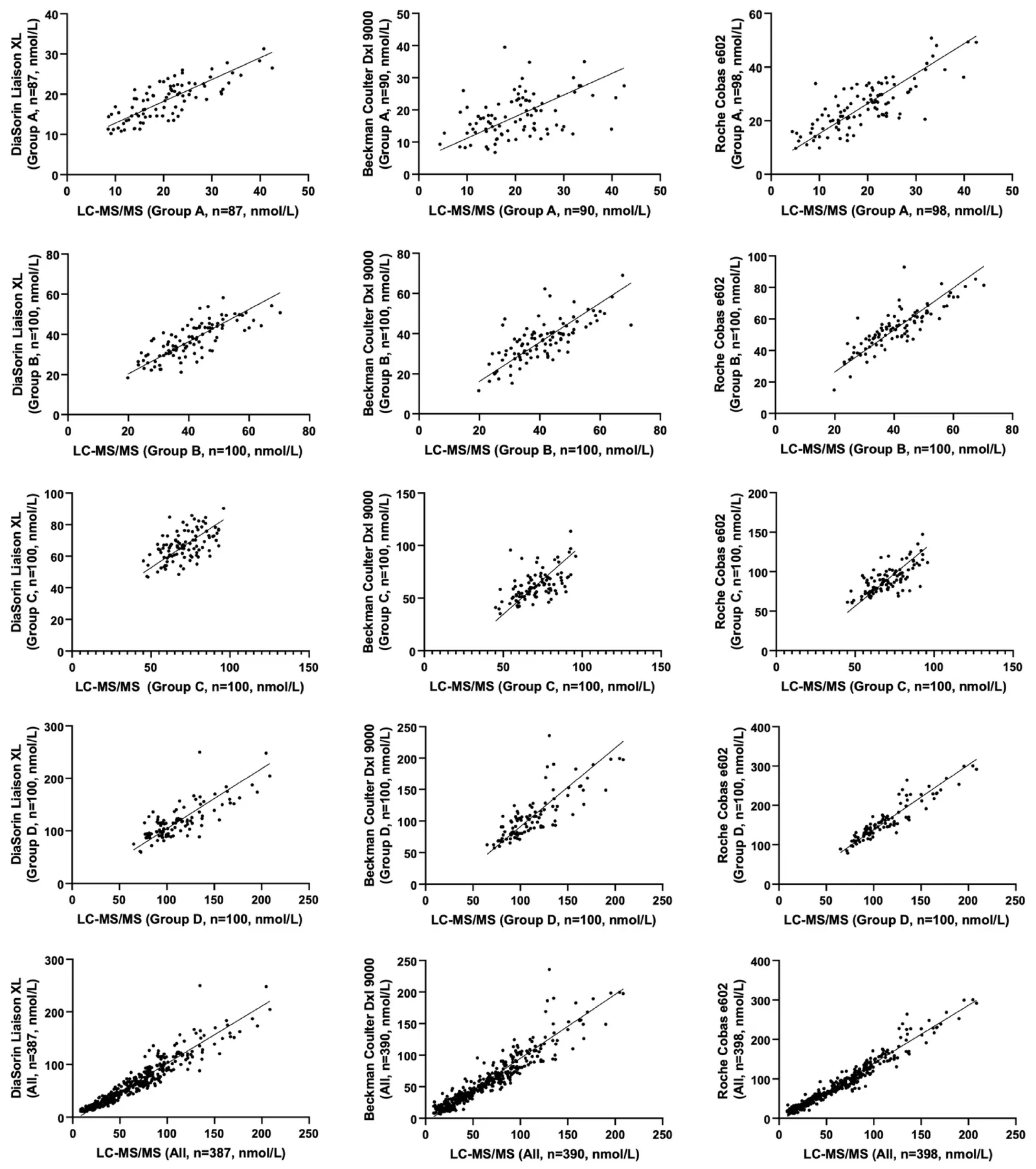
Deming regression plots comparing 25-hydroxyvitamin D concentrations measured by the three automated immunoassays with those measured by LC-MS/MS.

**Figure 4:**
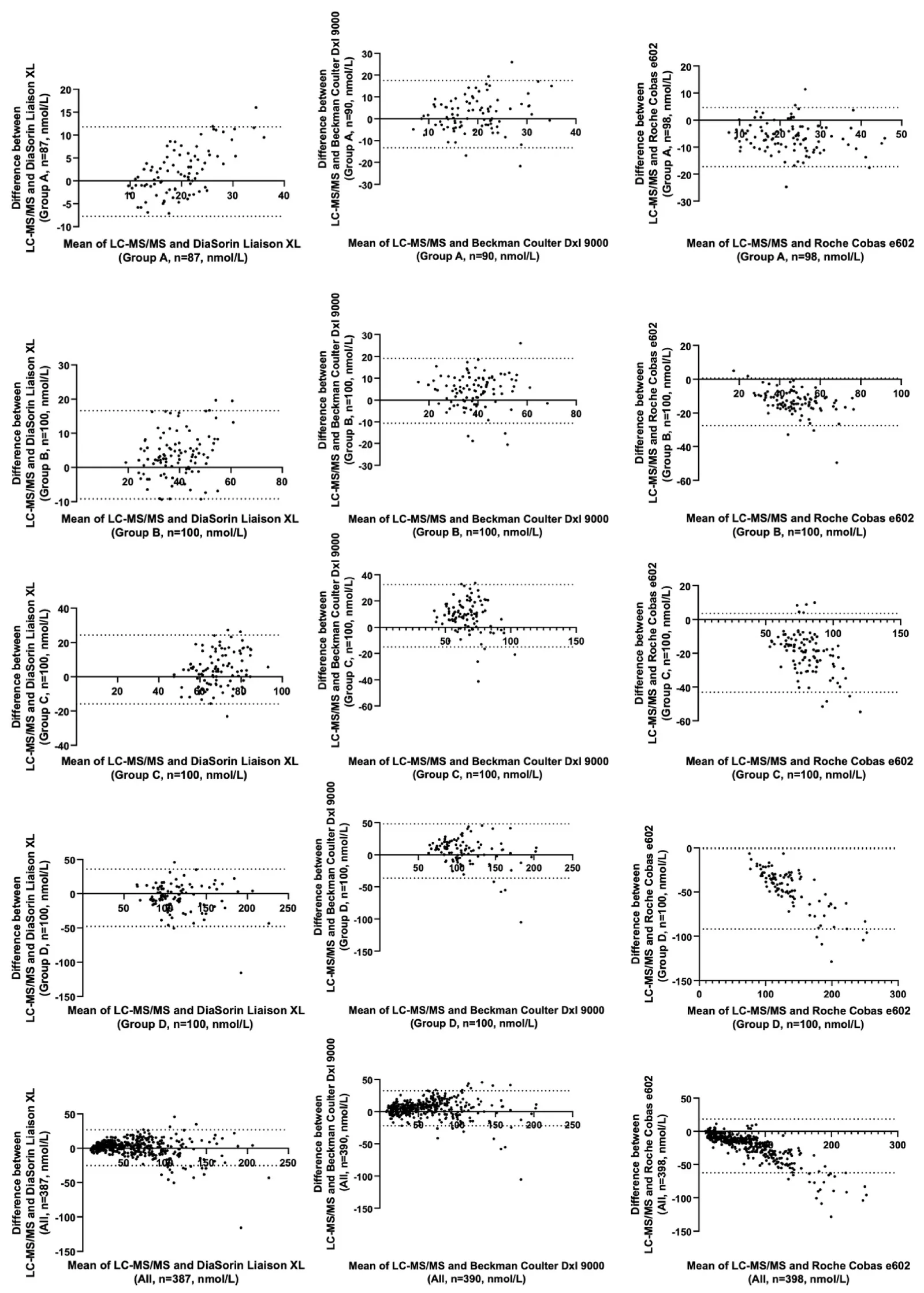
Bland–Altman plots showing agreement between the three automated immunoassays and the reference LC-MS/MS method for 25-hydroxyvitamin D measurements. For all Bland–Altman analyses, the paired difference was calculated as: Difference = Method A − Method B. For comparisons between the LC-MS/MS method and the DiaSorin Liaison XL, Beckman Coulter or Roche methods, LC-MS/MS was consistently designated as Method A. The horizontal dashed lines above and below the zero baseline represent the upper and lower Limits of Agreement (LoA), respectively.

The results of the Deming regression and Bland–Altman analyses comparing the three automated immunoassays with the reference LC-MS/MS method are summarized in Table 4 and illustrated in Figure 3 and Figure 4. Analyses were performed for each of the four predefined 25OHD concentration subgroups (Groups A–D) as well as for the overall study cohort. Samples with concentrations below the respective analytical measuring ranges were excluded from the Bland–Altman and Deming regression analyses. Accordingly, 13, 10, and 2 sample results were excluded from the DiaSorin Liaison XL (n = 387), Beckman Coulter DxI 9000 (n = 390), and Roche cobas e602 groups (n = 398), respectively. Statistically significant results are highlighted in Table 4.

Compared with LC-MS/MS, the Roche cobas e602 platform demonstrated a statistically significant negative bias, overestimating serum 25OHD concentrations in all subgroups except Group A (<25 nmol/L). Deming regression analysis of the DiaSorin Liaison XL platform revealed statistically significant constant and proportional bias across the entire measurement range. In contrast, the Beckman Access platform showed good overall agreement with LC-MS/MS, with a statistically significant positive bias observed only in Group A, comprising samples with severe vitamin D deficiency.

### 3.4. Effect of Analytical Method on Clinical Classification Based on the 25OHD Decision Threshold

**Figure 5:**
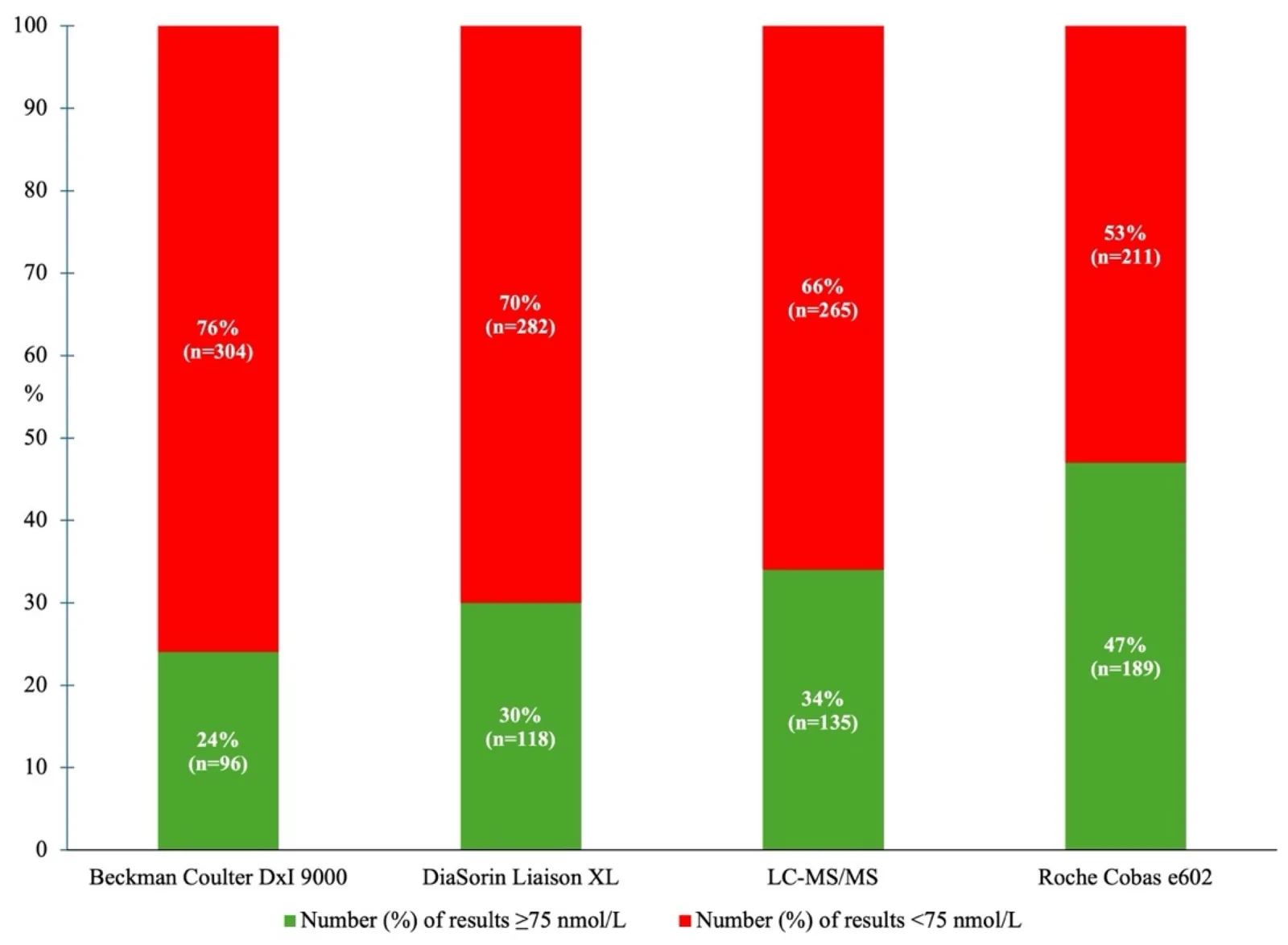
Percentage of samples classified as vitamin D sufficient (≥75 nmol/L) and insufficient (<75 nmol/L) by the four analytical methods.

The distribution of serum 25-hydroxyvitamin D (25OHD) results according to the clinically relevant decision threshold of 75 nmol/L is presented in Table 5 and Figure 5. For each analytical method, samples were classified as vitamin D sufficient (≥75 nmol/L) or insufficient (<75 nmol/L).

The proportion of samples classified as vitamin D insufficient (<75 nmol/L) varied across the four analytical methods. The Beckman Access DxI 9000 platform yielded the highest proportion of samples classified as insufficient, whereas the Roche cobas e602 platform yielded the lowest proportion (Table 5 and Figure 5), reflecting the methodological differences observed in the comparative analyses.

### 3.5. Diagnostic Performance at Clinically Relevant 25OHD Thresholds

Diagnostic classification performance was evaluated using LC-MS/MS as the reference method at the clinically relevant thresholds for severe vitamin D deficiency (<25 nmol/L), vitamin D deficiency (<50 nmol/L), and vitamin D insufficiency (<75 nmol/L). Confusion matrix analyses and diagnostic performance metrics for each threshold are presented below.

#### 3.5.1. Diagnostic Performance at the Severe Vitamin D Deficiency Threshold (<25 nmol/L)

Using LC-MS/MS as the reference method, 85 of the 400 samples were classified as having severe vitamin D deficiency (25OHD < 25 nmol/L).

The DiaSorin Liaison XL assay demonstrated the highest sensitivity (94.1%; 95% CI, 86.8–98.1), correctly identifying 80 of the 85 deficient samples, with five false-negative results. The Beckman Access assay showed a sensitivity of 90.6% (95% CI, 82.3–95.8), whereas the Roche cobas e602 assay exhibited a lower sensitivity of 61.2% (95% CI, 50.0–71.6). However, the Roche assay demonstrated the highest specificity (98.7%; 95% CI, 96.8–99.7), with only four false-positive classifications.

Agreement with the reference method was highest for the DiaSorin Liaison XL assay (Cohen’s κ = 0.818; 95% CI, 0.751–0.886), followed by the Beckman Access assay (κ = 0.795; 95% CI, 0.723–0.867) and the Roche cobas e602 assay (κ = 0.684; 95% CI, 0.587–0.781).

Among the three immunoassays, the Roche cobas e602 assay achieved the highest positive predictive value (92.9%; 95% CI, 82.7–98.0%), whereas the DiaSorin Liaison XL assay demonstrated the highest negative predictive value (98.3%; 95% CI, 96.1–99.5%).

McNemar’s test demonstrated statistically significant disagreement between each immunoassay and the reference LC-MS/MS method (all *p* < 0.05).

The confusion matrices and corresponding diagnostic performance metrics are presented in Table 6 and Table 7, respectively.

#### 3.5.2. Diagnostic Performance at the Vitamin D Deficiency Threshold (<50 nmol/L)

Using LC-MS/MS as the reference method and a clinical threshold of <50 nmol/L, 189 of the 400 samples were classified as vitamin D deficient.

The Beckman Access assay demonstrated the highest sensitivity (98.4%; 95% CI, 95.4–99.7), correctly identifying 186 of the 189 deficient samples and missing only three cases. The DiaSorin Liaison XL assay showed comparable performance, with a sensitivity of 97.4% (95% CI, 93.9–99.1). In contrast, the Roche cobas e602 assay detected only 75.7% of deficient samples (95% CI, 68.9–81.6), resulting in 46 false-negative classifications.

The Roche cobas e602 assay achieved perfect specificity (100.0%; 95% CI, 98.3–100.0) and a positive predictive value of 100.0% (95% CI, 97.5–100.0), indicating that all Roche-positive results were confirmed by LC-MS/MS. However, the DiaSorin Liaison XL and Beckman Access assays demonstrated substantially higher negative predictive values of 97.5% and 98.4%, respectively.

Overall agreement with LC-MS/MS was highest for the DiaSorin Liaison XL assay (Cohen’s κ = 0.895; 95% CI, 0.851–0.939), which also achieved the highest diagnostic accuracy (94.8%; 95% CI, 92.1–96.7). The Beckman Access assay likewise demonstrated almost perfect agreement with the reference method, whereas the Roche cobas e602 assay showed substantial agreement.

McNemar’s test demonstrated statistically significant disagreement between each immunoassay and LC-MS/MS (all *p* < 0.05), although the degree of discordance was smallest for the DiaSorin Liaison XL assay.

The confusion matrices and the corresponding diagnostic performance metrics are presented in Table 8 and Table 9, respectively.

#### 3.5.3. Diagnostic Performance at the Vitamin D Insufficiency Threshold (<75 nmol/L)

Using LC-MS/MS as the reference method and a clinical threshold of <75 nmol/L, 265 of the 400 samples were classified as vitamin D insufficient.

The Beckman Access assay demonstrated the highest sensitivity (98.1%; 95% CI, 95.7–99.4), closely followed by the DiaSorin Liaison XL assay (97.7%; 95% CI, 95.2–99.2). In contrast, the Roche cobas e602 assay exhibited substantially lower sensitivity (79.3%; 95% CI, 74.0–84.0), failing to identify 55 samples classified as insufficient by LC-MS/MS.

The Roche cobas e602 assay demonstrated the highest specificity (98.5%; 95% CI, 94.7–99.8), generating only two false-positive results. The DiaSorin Liaison XL assay maintained good specificity (82.8%; 95% CI, 75.4–88.8), whereas the Beckman Access assay showed the lowest specificity (67.2%; 95% CI, 58.5–75.0), producing 44 false-positive classifications.

The Roche cobas e602 assay achieved the highest positive predictive value (99.1%; 95% CI, 96.6–99.9), indicating that nearly all Roche-positive results were confirmed by LC-MS/MS. The DiaSorin Liaison XL assay provided the most balanced diagnostic performance, with a PPV of 91.9% (95% CI, 88.1–94.8) and a negative predictive value of 94.9% (95% CI, 89.2–98.1). Both the DiaSorin Liaison XL and Beckman Access assays demonstrated excellent NPVs (>94%).

Overall agreement with the reference method was highest for the DiaSorin Liaison XL assay (Cohen’s κ = 0.832; 95% CI, 0.773–0.891), which also achieved the highest overall diagnostic accuracy (92.8%; 95% CI, 89.8–95.1).

McNemar’s test demonstrated statistically significant disagreement between each immunoassay and LC-MS/MS (all *p* < 0.05).

The confusion matrices and the corresponding diagnostic performance metrics are presented in Table 10 and Table 11, respectively.

### 3.6. Effect of Vitamin D Metabolites on 25-Hydroxyvitamin D Measurements by the Three Automated Immunoassays

To investigate the influence of vitamin D metabolites on serum 25-hydroxyvitamin D (25OHD) measurements obtained with the three automated immunoassays, correlation analyses were performed between the measurement bias (defined as the difference between LC-MS/MS and immunoassay-derived 25OHD concentrations) and the corresponding concentrations of the vitamin D metabolites C3-epi-25-hydroxyvitamin D_3_ and 24,25-dihydroxyvitamin D_3_. The LC-MS/MS-derived metabolite concentrations are summarized in Table 12.

For the Roche cobas e602 assay, statistically significant negative correlations were observed between measurement bias and both vitamin D metabolites: C3-epi-25-hydroxyvitamin D_3_ (Spearman’s ρ = −0.6141; 95% CI, −0.7123 to −0.4923) and 24,25-dihydroxyvitamin D_3_ (Spearman’s ρ = −0.5595; 95% CI, −0.6687 to −0.4267). For the DiaSorin Liaison XL assay, a statistically significant negative correlation was only observed with 24,25-dihydroxyvitamin D_3_ (Spearman’s ρ = −0.3657; 95% CI, −0.5078 to −0.2042). No statistically significant correlations were observed for the Beckman Access assay. Since C3-epi-25-hydroxyvitamin D3 and especially 24,25-dihydroxyvitamin D3 are related to total 25OHD concentrations, the present correlation analyses cannot establish independent analytical interference.

**Figure 6:**
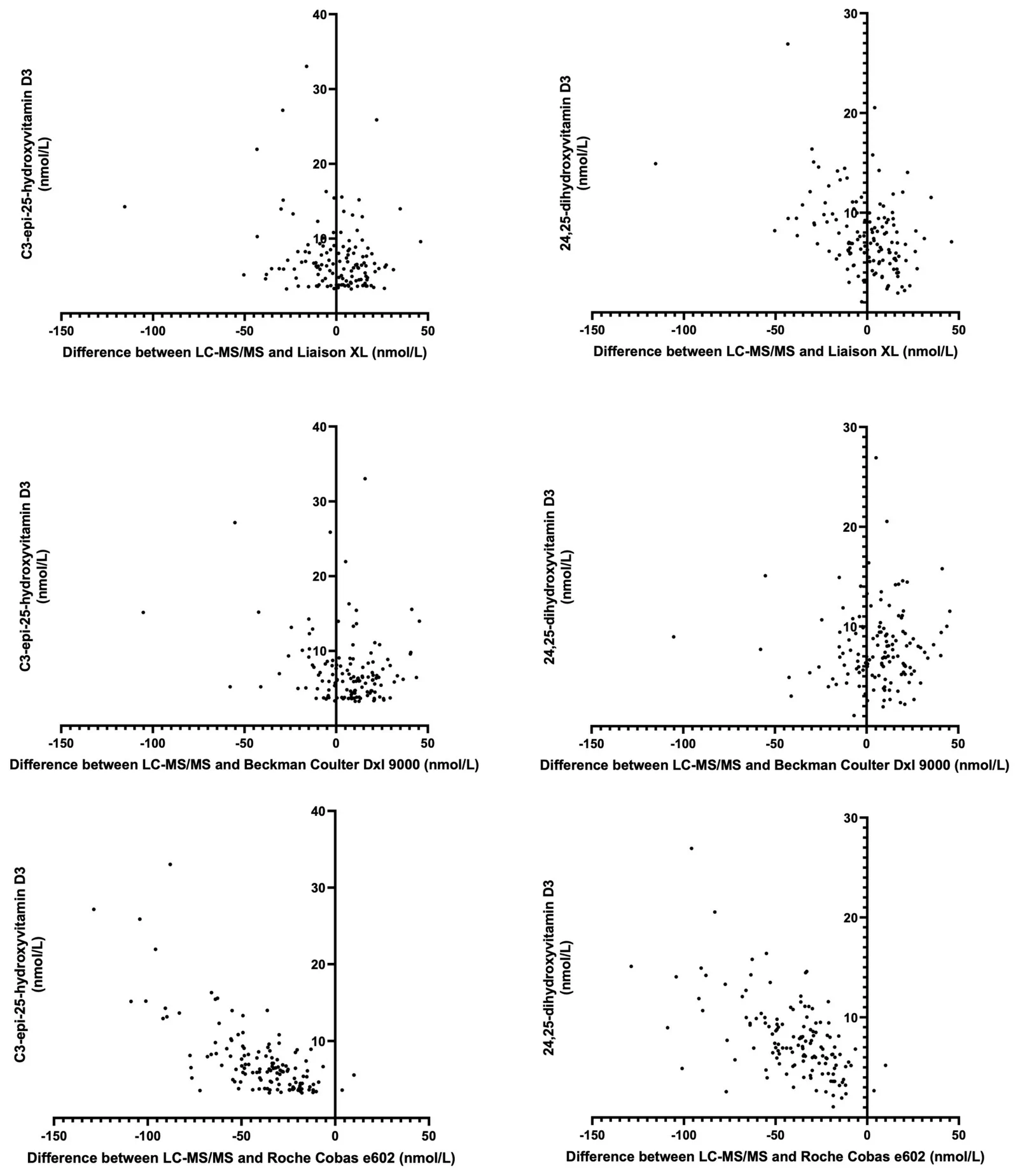
Correlation between serum vitamin D metabolite concentrations and the measurement bias (LC-MS/MS minus immunoassay) for the three automated immunoassays.

The relationships between the vitamin D metabolites and the measurement bias for each immunoassay are illustrated in Figure 6.

### 3.7. External Quality Assurance

**Figure 7:**
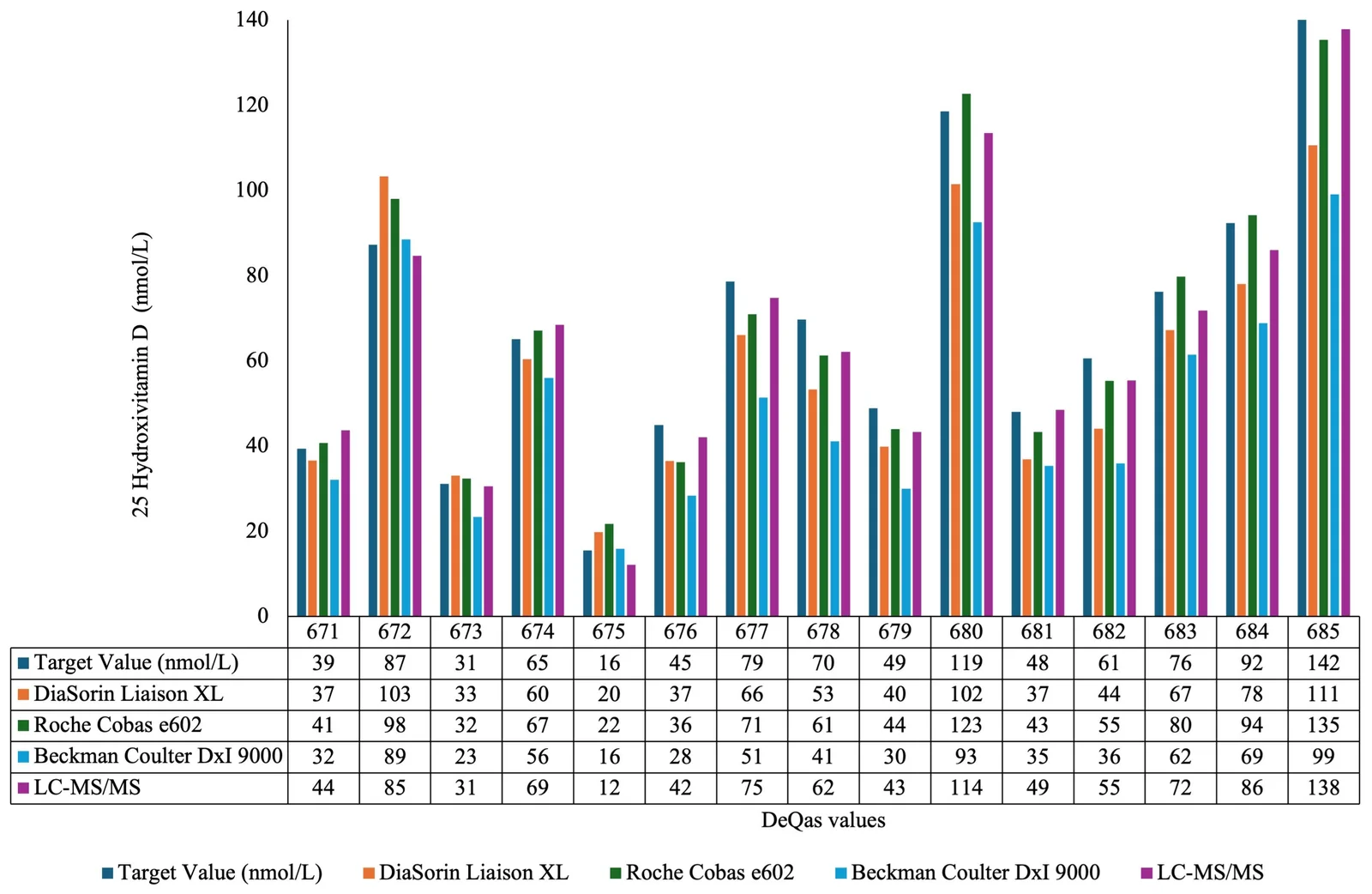
Distribution of 25OHD concentrations measured in DEQAS samples 671–685 using the four analytical methods.

Throughout the study period, all four analytical platforms participated in the DEQAS external quality assessment program. All methods exhibited both positive and negative deviations from the assigned target values. Overall, predominantly negative biases were observed for the DiaSorin Liaison XL, Beckman Coulter immunoassays, and the LC-MS/MS method, whereas the Roche assay showed a balanced distribution of positive and negative deviations. The magnitude of bias varied across samples and methods (Table 13, Figure 7).

According to DEQAS performance criteria, at least 75% of 25-hydroxyvitamin D (25OHD) results must fall within ±25% of the assigned target value to achieve certification. This criterion was met by the LC-MS/MS, DiaSorin Liaison XL, and Roche methods but not by the Beckman Coulter assay.

## 4. Discussion

The present study provides a comprehensive comparison of three widely used automated 25-hydroxyvitamin D immunoassays against a reference LC-MS/MS method using a deliberately balanced cohort covering the clinically relevant concentration ranges of severe deficiency, deficiency, insufficiency and sufficiency. Unlike many previous comparison studies, the present work also evaluated the influence of long-term sample storage, vitamin D metabolites and external quality assessment performance, thereby providing a more complete assessment of assay performance under routine laboratory conditions.

One of the major findings was the marked variability between immunoassays despite all methods measuring the same analyte, despite accounting for the effect of storage at −70 °C. The data suggest that DiaSorin Liaison XL is closer to LC-MS/MS for some criteria, for example the overall proportion of samples classified ≥75 nmol/L or below 75 nmol/L, but the Deming regression still indicates a statistically significant constant and proportional deviation across the measurement range. In addition, the relative performance of the assays appears to depend on the criterion considered, including mean bias, limits of agreement, clinical classification, DEQAS results, and potential influence of vitamin D metabolites. Compared with LC-MS/MS, Roche Cobas e602 produced higher 25OHD values overall and tended to classify fewer samples as deficient or insufficient when the <25, <50 and <75 nmol/L thresholds were applied. The authors acknowledge that no universal consensus has yet been established regarding the thresholds defining vitamin D sufficiency. For the detection of samples below these thresholds, this resulted in lower sensitivity and a higher number of false-negative classifications, despite high specificity. Beckman Coulter tended to generate lower values than LC-MS/MS and showed more false-positive classifications, particularly at the <75 nmol/L threshold. DiaSorin Liaison XL showed the best overall balance between sensitivity, specificity, accuracy and agreement with LC-MS/MS across the three clinical thresholds, although significant analytical discrepancies remained. These observations are in agreement with previous comparison studies demonstrating that method-dependent bias remains an important limitation of automated vitamin D immunoassays despite ongoing standardization efforts [29,30,31,32,33,34,35,36,37,38,39].

The balanced study design also allowed evaluation of assay performance within clinically relevant decision thresholds. This is particularly important because laboratory agreement should ultimately be judged according to its impact on patient classification rather than analytical agreement alone. DiaSorin Liaison XL consistently showed the highest overall agreement with LC-MS/MS across all three clinical thresholds, whereas Beckman Coulter demonstrated the highest sensitivity for detecting vitamin D deficiency but at the expense of lower specificity, especially at the 75 nmol/L threshold. Conversely, Roche exhibited excellent specificity and positive predictive value but substantially lower sensitivity owing to a higher number of false-negative classifications. These findings indicate that assay selection may influence clinical decision-making, particularly when vitamin D status is close to established diagnostic cut-offs.

The potential influence of vitamin D metabolites, particularly C3-epi-25-hydroxyvitamin D3 and 24,25-dihydroxyvitamin D3, has previously been highlighted by the DEQAS group and other studies [28,41]. In the present study, higher concentrations of these metabolites were associated with a larger positive deviation in Roche cobas e602 results compared with LC-MS/MS, as reflected by the increasingly negative LC-MS/MS minus Roche difference. A weaker association was also observed for DiaSorin Liaison XL and 24,25-dihydroxyvitamin D3, whereas Beckman Coulter results did not appear to be influenced in the same way. These findings suggest that metabolite-related effects may contribute to method-specific differences, although this interpretation should remain cautious because 24,25-dihydroxyvitamin D3 is strongly related to total 25OHD concentration. Additional analyses adjusted for total 25OHD would be useful to distinguish true analytical interference from concentration-dependent bias. These findings support previous observations from the DEQAS Working Group suggesting that incomplete analytical specificity toward structurally related vitamin D metabolites remains an important contributor to inter-method variability [41]. The present study confirms these observations under real-world clinical conditions using routine patient samples.

Based on the results of 15 DEQAS samples, performance in the external quality assessment (EQA) (DEQAS) did not fully mirror the findings obtained in the patient samples. While Roche demonstrated acceptable external quality assessment performance, the same platform showed systematic overestimation relative to LC-MS/MS in the present study. Conversely, Beckman Coulter failed to achieve DEQAS certification despite exhibiting good agreement with LC-MS/MS in the patient cohort. These discrepancies may reflect differences between EQA materials and routine patient samples, including matrix effects, sample composition and metabolite concentrations. Therefore, DEQAS results should be interpreted as complementary to, rather than interchangeable with, patient-sample comparison studies. These findings suggest that external quality assessment performance does not necessarily predict clinical agreement with reference measurement procedures and highlights the importance of evaluating assay performance using both proficiency testing materials and authentic patient samples.

The present study has several strengths, including the relatively large sample size, equal representation across clinically important vitamin D categories, simultaneous comparison of three major automated immunoassays, evaluation of storage effects, investigation of vitamin D metabolites and inclusion of DEQAS performance. Nonetheless, several limitations of the present study should be acknowledged. First, the study employed an artificially balanced distribution of samples across four predefined vitamin D concentration categories, with initial sample selection based on DiaSorin Liaison XL assay results. This enriched study design may have introduced spectrum and selection bias and may have influenced estimates of diagnostic performance, including positive predictive value (PPV), negative predictive value (NPV), and overall accuracy, as well as comparisons between the evaluated analytical platforms. Accordingly, these performance measures should be interpreted within the context of the study design and should not be considered directly representative of an unselected routine clinical population. Further studies using consecutively collected, clinically representative patient samples with a naturally occurring distribution of vitamin D concentrations would be valuable to confirm the generalisability of the present findings. Additionally, the study was conducted at a single centre, only adult outpatient samples were included, and all specimens originated from one geographical region. Furthermore, only one LC-MS/MS reference laboratory was used, although this laboratory employs a validated reference methodology.

Overall, the present findings demonstrate that clinically relevant differences remain between automated vitamin D immunoassays, despite ongoing international standardization initiatives. Laboratories and clinicians should therefore interpret vitamin D results with awareness of the analytical method employed, particularly when treatment decisions rely on established clinical decision thresholds.

## 5. Conclusions

The present study indicates that analytical and clinical differences persist among automated 25-hydroxyvitamin D immunoassays despite ongoing international standardization efforts. DiaSorin Liaison XL showed the closest overall agreement with LC-MS/MS across the evaluated clinical decision thresholds, whereas Beckman Coulter demonstrated high sensitivity for detecting vitamin D deficiency but lower specificity at higher thresholds. Roche cobas tended to overestimate 25OHD concentrations, with correspondingly lower sensitivity but higher specificity and positive predictive value. Acknowledging that the present correlation analyses cannot establish independent analytical interference, the observed influence of vitamin D metabolites, particularly for Roche and to a lesser extent DiaSorin, may emphasize the importance of assay-specific analytical characteristics. These findings suggest that assay selection may affect patient classification and clinical interpretation. Continued assay harmonization and awareness of platform-specific performance characteristics are therefore important for improving the comparability and clinical interpretation of 25OHD measurements in routine practice.

## Figures and Tables

**Table 1:** Major features of the automated 25-hydroxyvitamin D assays included in the study.

Sample Group	DiaSorin Liaison 25 OH Vitamin D Total Assay	Beckman Access 25(OH) Vitamin D Total Assay	Roche Elecsys Vitamin D Total III Assay
Instrument	Liaison XL automated immunoassay analyzer	DxI 9000 Access immunoassay analyzer	cobas e602 analyzer
Reagent catalogue number	310600	B24838	09038078190
Reagent LOT number	137483	572136	869010
Analytical range, nmol/L	10–375	11–418	7.5–300
Standardization to VDSP	Yes	Yes	Yes
Within-run precision, CV%	4.8–7.7	1.5–3.8	2.3–7.4
Total precision, CV%	8.8–12.5	6.8–7.7	3.3–9.8

**Table 2:** Comparison of serum 25-hydroxyvitamin D concentrations measured by the DiaSorin Liaison XL platform before and after storage at −70 °C.

Sample Group	Before Storage (Mean, Range)	After Storage (Mean, Range)	% Difference (Mean, Range)	*p* Value
A (<25 nmol/L)	18 (<10–24) nmol/L	17 (<10–31) nmol/L	−9 (−79 to +34)%	<0.001
B (25–49 nmol/L)	38 (25–49) nmol/L	37 (18–58) nmol/L	−2 (−31 to +35)%	0.189
C (50–74 nmol/L)	63 (50–74) nmol/L	67 (47–90) nmol/L	+5 (−16 to +32)%	<0.001
D (≥75 nmol/L)	108 (75–222) nmol/L	119 (59–250) nmol/L	+9 (−27 to +51)%	<0.001
All (n = 400)	57 (<10–222) nmol/L	60 (<10–250) nmol/L	+1 (−79 to +51%)	<0.001

**Table 3:** Summary statistics of serum 25-hydroxyvitamin D concentrations measured using the four analytical platforms.

Sample Group	DiaSorin Liaison XL (Mean, Range)	Beckman Coulter (Mean, Range)	Roche (Mean, Range)	LC-MS/MS (Mean, Range)
A (<25 nmol/L)	17 (<10–31) nmol/L	17 (<11–40) nmol/L	25 (<7.5–50) nmol/L	19 (<4.4–43) nmol/L
B (25–49 nmol/L)	37 (18–58) nmol/L	37 (12–69) nmol/L	54 (15–93) nmol/L	41 (20–70) nmol/L
C (50–74 nmol/L)	67 (47–90) nmol/L	62 (35–114) nmol/L	90 (61–147) nmol/L	71 (45–96) nmol/L
D (≥75 nmol/L)	119 (59–250) nmol/L	107 (57–236) nmol/L	159 (79–301) nmol/L	113 (65–209) nmol/L
All (n = 400)	60 (<10–250) nmol/L	56 (<11–236) nmol/L	82 (<7.5–301) nmol/L	61 (<4.4–209)

**Table 4:** Results of the Deming regression and Bland–Altman analyses comparing the three automated immunoassays with the reference LC-MS/MS method.

	Deming Regression		Bland–Altman
Immunoassay (Group)	Slope (95% CI)	Y-Intercept (95% CI)	Bias (95% LoA)
DiaSorin Liaison XL (All)	1.089 (1.019 to 1.158)	−6.589 (−10.11 to −3.066)	1.007 (−25.10 to 27.11)
DiaSorin Liaison XL (Group A)	0.5422 (0.458 to 0.626)	7.389 (5.595 to 9.183)	2.032 (−7.735 to 11.80)
DiaSorin Liaison XL (Group B)	0.803 (0.663 to 0.943)	4.323 (−1.231 to 9.877)	3.727 (−9.175 to 16.63)
DiaSorin Liaison XL (Group C)	0.661 (0.488 to 0.834)	19.72 (7.667 to 31.77)	4.229 (−15.91 to 24.37)
DiaSorin Liaison XL (Group D)	1.145 (0.864 to 1.425)	−10.43 (−39.81 to 18.94)	−5.890 (−47.51 to 35.73)
Beckman Coulter (All)	1.012 (0.940 to 1.084)	−6.161 (−9.861 to −2.461)	5.397 (−21.68 to 32.47)
Beckman Coulter (A)	0.672 (0.352 to 0.992)	4.489 (−1.514 to 10.49)	2.153 (−13.26 to 17.57)
Beckman Coulter (B)	0.9738 (0.775 to 1.172)	−3.227 (−10.99 to 4.534)	4.298 (−10.58 to 19.18)
Beckman Coulter (C)	1.320 (0.939 to 1.700)	−31.38 (−57.26 to −5.492)	8.753 (−14.84 to 32.35)
Beckman Coulter (D)	1.250 (0.970 to 1.530)	−33.84 (−62.50 to −5.188)	5.616 (−36.61 to 47.84)
Roche (All)	1.470 (1.418 to 1.522)	−7.551 (−10.31 to −4.796)	−22.02 (−62.54 to 18.49)
Roche (A)	1.119 (0.967 to 1.270)	3.981 (1.068 to 6.895)	−6.267 (−17.21 to 4.677)
Roche (B)	1.333 (1.110 to 1.556)	−0.249 (−8.950 to 8.451)	−13.35 (−27.38 to 0.649)
Roche (C)	1.645 (1.351 to 1.939)	−25.94 (−46.56 to −5.323)	−19.68 (−43.01 to 3.646)
Roche (D)	1.650 (1.495 to 1.805)	−26.98 (−42.60 to −11.35)	−46.41 (−91.97 to −0.844)

The shaded values are statistically significant (*p* < 0.05). CI: Confidence of Interval, LoA: Limit of Agreement. For all Bland–Altman analyses, the paired difference was calculated as: Difference = Method A − Method B. For comparisons between the LC-MS/MS method and the DiaSorin Liaison XL, Beckman Coulter or Roche methods, LC-MS/MS was consistently designated as Method A.

**Table 5:** Distribution of serum 25-hydroxyvitamin D results according to the clinical decision threshold of 75 nmol/L measured by the four analytical methods.

Method	Number (%) of Results ≥ 75 nmol/L	Number (%) of Results < 75 nmol/L
DiaSorin Liaison XL	118 (30%)	282 (70%)
Beckman Coulter	96 (24%)	304 (76%)
Roche	189 (47%)	211 (53%)
LC-MS/MS	135 (34%)	265 (66%)

**Table 6:** Confusion matrices comparing the three automated immunoassays with the reference LC-MS/MS method for the detection of severe vitamin D deficiency (25OHD < 25 nmol/L).

	True Positive (n, %)	False Positive (n, %)	True Negative (n, %)	False Negative (n, %)
DiaSorin Liaison XL	80 (20%)	21 (5.25%)	294 (73.5%)	5 (1.25%)
Beckman Coulter	77 (19.25%)	21 (5.25%)	294 (73.5%)	8 (2%)
Roche	52 (13%)	4 (1%)	311 (77.75%)	33 (8.25%)

**Table 7:** Diagnostic performance of the three automated immunoassays compared with LC-MS/MS for the detection of severe vitamin D deficiency (25OHD < 25 nmol/L).

	DiaSorin Liaison XL	Beckman Coulter	Roche
Sensitivity % (95% CI)	94.1 (86.8–98.1)	90.6 (82.3–95.8)	61.2 (50.0–71.6)
Specificity % (95% CI)	93.3 (90.0–95.8)	93.3 (90.0–95.8)	98.7 (96.8–99.7)
PPV % (95% CI)	79.2 (70.0–86.6)	78.6 (69.1–86.2)	92.9 (82.7–98.0)
NPV % (95% CI)	98.3 (96.1–99.5)	97.4 (94.8–98.8)	90.4 (86.8–93.3)
Accuracy % (95% CI)	93.5 (90.6–95.7)	92.8 (89.8–95.1)	90.8 (87.5–93.4)
Cohen’s κ (95% CI)	0.818 (0.751–0.886)	0.795 (0.723–0.867)	0.684 (0.587–0.781)
McNemar *p*	0.0025	0.0241	<0.0001

Sensitivity, specificity, positive predictive value (PPV), negative predictive value (NPV) and accuracy CIs are exact Clopper–Pearson (binomial) 95% confidence intervals.

**Table 8:** Confusion matrices comparing the three automated immunoassays with the reference LC-MS/MS method for the detection of vitamin D deficiency (25OHD < 50 nmol/L).

	True Positive (n, %)	False Positive (n, %)	True Negative (n, %)	False Negative (n, %)
DiaSorin Liaison XL	184 (46%)	16 (4%)	195 (48.8%)	5 (1.3%)
Beckman Coulter	186 (46.5%)	24 (6%)	187 (46.8%)	3 (0.8%)
Roche	143 (35.8%)	0 (0%)	211 (52.8%)	46 (11.5%)

**Table 9:** Diagnostic performance of the three automated immunoassays compared with LC-MS/MS for the detection of vitamin D deficiency (25OHD < 50 nmol/L).

	DiaSorin Liaison XL	Beckman Coulter	Roche
Sensitivity % (95% CI)	97.4 (93.9–99.1)	98.4 (95.4–99.7)	75.7 (68.9–81.6)
Specificity % (95% CI)	92.4 (88.0–95.6)	88.6 (83.5–92.6)	100.0 (98.3–100.0)
PPV % (95% CI)	92.0 (87.3–95.4)	88.6 (83.5–92.5)	100.0 (97.5–100.0)
NPV % (95% CI)	97.5 (94.3–99.2)	98.4 (95.5–99.7)	82.1 (76.9–86.6)
Accuracy % (95% CI)	94.8 (92.1–96.7)	93.3 (90.3–95.5)	88.5 (85.0–91.5)
Cohen’s κ (95% CI)	0.895 (0.851–0.939)	0.865 (0.816–0.914)	0.766 (0.703–0.830)
McNemar *p*	0.0266	<0.0001	<0.0001

Sensitivity, specificity, positive predictive value (PPV), negative predictive value (NPV) and accuracy CIs are exact Clopper–Pearson (binomial) 95% confidence intervals.

**Table 10:** Confusion matrices comparing the three automated immunoassays with the reference LC-MS/MS method for the detection of vitamin D insufficiency (25OHD < 75 nmol/L).

	True Positive (n, %)	False Positive (n, %)	True Negative (n, %)	False Negative (n, %)
DiaSorin Liaison XL	260 (65%)	23 (5.8%)	111 (27.8%)	6 (1.5%)
Beckman Coulter	261 (65.3%)	44 (11%)	90 (22.5%)	5 (1.3%)
Roche	211 (52.8%)	2 (0.5%)	132 (33%)	55 (13.8%)

**Table 11:** Diagnostic performance of the three automated immunoassays compared with LC-MS/MS for the detection of vitamin D insufficiency (25OHD < 75 nmol/L).

Assay	DiaSorin Liaison XL	Beckman Coulter	Roche
Sensitivity % (95% CI)	97.7 (95.2–99.2)	98.1 (95.7–99.4)	79.3 (74.0–84.0)
Specificity % (95% CI)	82.8 (75.4–88.8)	67.2 (58.5–75.0)	98.5 (94.7–99.8)
PPV % (95% CI)	91.9 (88.1–94.8)	85.6 (81.1–89.3)	99.1 (96.6–99.9)
NPV % (95% CI)	94.9 (89.2–98.1)	94.7 (88.1–98.3)	70.6 (63.5–77.0)
Accuracy % (95% CI)	92.8 (89.8–95.1)	87.8 (84.1–90.8)	85.8 (81.9–89.0)
Cohen’s κ (95% CI)	0.832 (0.773–0.891)	0.704 (0.626–0.781)	0.709 (0.639–0.779)
McNemar *p*	0.0023	<0.0001	<0.0001

Sensitivity, specificity, positive predictive value (PPV), negative predictive value (NPV) and accuracy CIs are exact Clopper–Pearson (binomial) 95% confidence intervals.

**Table 12:** Mean concentrations of C3-epi-25-hydroxyvitamin D_3_ and 24,25-dihydroxyvitamin D_3_, as determined by LC-MS/MS, in the study samples.

Sample Group	25-Hydroxyvitamin D_2_ (Mean, Range)	25-Hydroxyvitamin D_3_ (Mean, Range)	Total 25-Hydroxyvitamin D (Mean, Range)	C3-epi-25-Hydroxyvitamin D_3_ (Mean, Range)	24,25-Dihydroxyvitamin D_3_ (Mean, Range)
A (<25 nmol/L)	4.6 (<4.4–11)	18.2 (4–41)	19 (4–43)	<3.25	0.8 (<0.62–2.7)
B (25–49 nmol/L)	4.8 (4.4–15)	39.6 (19.7–65.4)	40.9 (19.8–70.3)	3.3 (<3.25–8.9)	2.2 (0.7–4.4)
C (50–74 nmol/L)	4.4 (4.25–8.6)	70.4 (45–96)	70.7 (45–96)	3.8 (<3.25–8.7)	4.7 (1.7–8.1)
D (≥75 nmol/L)	4.5 (<4.4–9.5)	112.3 (65–208.6)	113 (65–208.6)	8.2 (<3.25–33)	9 (2.6–27)
All (n = 400)	4.6 (<4.4–15)	60.1 (4–208.6)	61 (4–208.6)	4.6 (<3.25–33)	4.2 (<0.62–27)

All values are in nmol/L.

**Table 13:** DEQAS results and bias relative to assigned target values for the four analytical platforms.

2025 Cycle	Target Value (nmol/L)	DiaSorin Liaison XL		Roche Cobas e602		Beckman Coulter DxI 9000		LC-MS/MS	
		Our 25OHD (nmol/L)	Bias (%)	Our 25OHD (nmol/L)	Bias (%)	Our 25OHD (nmol/L)	Bias (%)	Our 25OHD (nmol/L)	Bias (%)
April									
671	39.4	36.6	−7.1	40.7	3.3	32.1	−18.5	43.7	10.9
672	87.3	103.3	18.3	98.0	12.3	88.5	1.4	84.7	−3.0
673	31.1	33.1	6.4	32.4	4.2	23.4	−24.8	30.6	−1.6
674	65.1	60.4	−7.2	67.1	3.1	56.0	−14.0	68.5	5.2
675	15.5	19.8	27.7	21.7	40.0	15.9	2.6	12.1	−21.9
July									
676	45.0	36.5	−18.9	36.2	−19.6	28.4	−36.9	42.1	−6.4
677	78.7	66.1	−16.0	71.0	−9.8	51.4	−34.7	74.8	−5.0
678	69.7	53.3	−23.5	61.3	−12.1	41.1	−41.0	62.1	−10.9
679	48.9	39.9	−18.4	44.0	−10.0	30.0	−38.7	43.3	−11.5
680	118.6	101.5	−14.4	122.7	3.5	92.6	−21.9	113.5	−4.3
October									
681	48.0	36.9	−23.1	43.3	−9.8	35.4	−26.3	48.5	1.0
682	60.6	44.1	−27.2	55.3	−8.7	35.9	−40.8	55.4	−8.6
683	76.3	67.2	−11.9	79.8	4.6	61.5	−19.4	71.8	−5.9
684	92.4	78.1	−15.5	94.2	1.9	68.9	−25.4	86.0	−6.9
685	142.3	110.6	−22.3	135.4	−4.8	99.1	−30.4	137.9	−3.1

## Data Availability

The data supporting the findings of this study are available from the corresponding author upon reasonable request. The data are not publicly available due to privacy restrictions.
